# Financial Risk Protection and Universal Health Coverage: Evidence and Measurement Challenges

**DOI:** 10.1371/journal.pmed.1001701

**Published:** 2014-09-22

**Authors:** Priyanka Saksena, Justine Hsu, David B. Evans

**Affiliations:** 1 Department of Health Systems Governance and Financing, World Health Organization, Geneva, Switzerland; 2 Swiss Tropical and Public Health Institute, University of Basel, Basel Switzerland

## Abstract

Financial risk protection is a key component of universal health coverage (UHC), which is defined as access to all needed quality health services without financial hardship. As part of the *PLOS Medicine* Collection on measurement of UHC, the aim of this paper is to examine and to compare and contrast existing measures of financial risk protection. The paper presents the rationale behind the methodologies for measuring financial risk protection and how this relates to UHC as well as some empirical examples of the types of measures. Additionally, the specific challenges related to monitoring inequalities in financial risk protection are discussed. The paper then goes on to examine and document the practical challenges associated with measurement of financial risk protection. This paper summarizes current thinking on the area of financial risk protection, provides novel insights, and suggests future developments that could be valuable in the context of monitoring progress towards UHC.

## Abstract 2

As part of a PLOS Collection on universal health coverage, Priyanka Saksena and colleagues examine existing measures of financial risk protection and suggest future developments that could be valuable in monitoring progress towards universal health coverage.

This paper is part of the PLOS Universal Health Coverage Collection.

**Key Summary Points**

- Health payments are a heavy financial burden for millions around the world. Financial risk protection is concerned with safeguarding people against the financial hardship associated with paying for health services.
- Two commonly applied concepts capture the lack of financial risk protection. The first, catastrophic health expenditure, occurs when a household's out-of-pocket (OOP) payments are so high relative to its available resources that the household foregoes the consumption of other necessary goods and services. The second concept, impoverishment, occurs when OOP payments push households below or further below the poverty line, a threshold under which even the most basic standard of living is not ensured.
- Headcount indicators, which measure the number of people affected, alone do not give the full picture of the problem. Additional measures of the intensity of financial hardship provide useful insights into the nature of OOP payments in different settings.
- Robust monitoring of financial risk protection requires reliable household expenditure surveys ideally conducted every 2 to 5 years.

## Financial Risk Protection and Universal Health Coverage

Health systems have developed specifically to allow people to use the health services they might need while protecting them against the adverse financial consequences of paying for care [1],[2]. This goal is now widely known as universal health coverage (UHC). It was the motivation for the social health insurance systems that developed in Europe, the National Health Service in the UK, and the recent reforms in the US now colloquially known as Obama care [3]–[5]. It is also the motivation for many of the recent adjustments to health systems and health financing systems in low- and middle-income countries [1].

Despite these developments, the burden imposed by out-of-pocket (OOP) health payments still results in financial hardship for millions of people who seek care globally [6]. OOP payments are also the most regressive form of financing for health. Thus UHC's focus on financial hardship arising from OOP payments is supported by on-the-ground realities.

But in a broader sense, financial hardship in UHC represents the impact of the health systems on the non-health aspects of people's lives. Households can be impoverished or be faced with catastrophic health expenditure from accessing needed health services. Fundamentally, the assurance that people will not suffer financial hardship in using services, an integral component of UHC, is a recognition that health systems should not only improve health but this improvement should not be done in ways that are detrimental to non-health aspects of well-being.

The concept of financial risk protection, or conversely the absence of a risk of financial hardship, has been the focus of interest to economists and researchers for many years, and measuring the ability of a health system to protect people against the financial hardship associated with paying for health services has become an important issue for research and analysis across countries at all income levels [6]–[11]. Many ways of measuring financial risk protection directly reflect the trade-offs people have to make between paying for the health services they need and paying for other necessities such as food and basic education [12],[13].

However, there has been considerable debate about whether the common measures actually capture the concept of the value of financial risk protection in itself, or whether they reflect the impact of the lack of financial protection—slightly different concepts, although both are commonly placed under the heading of financial risk protection [14],[15]. These discussions are particularly relevant as UHC gains prominence as a key international health systems goal.

In this context, this paper contributes to the discussion by providing a detailed analysis of key issues surrounding financial risk protection as a component of UHC. It examines the existing ways of monitoring UHC and the ideas underpinning them in non-technical language. The paper then considers the practical measurement challenges of using these methodologies. In this paper we summarize current thinking, provide novel insights, and suggest future developments that could be valuable in the context of monitoring progress towards UHC. Other papers in this Collection address the extent of financial risk protection in specific countries, so this paper does not include country-specific data analysis. Additionally, it should be noted that in this paper we do not discuss any targets for improving financial risk protection outcomes—instead we focus on measurement of financial risk protection.

## Some Underlying Principles behind Financial Risk Protection in Universal Health Coverage

The prominence of financial risk protection as a health systems' goal in itself and as an integral part of UHC partially arises because of its unique position as an interface between health systems and other dimensions of well-being. The absence of financial hardship in accessing health services means that the choice to use health services does not come at the price of poor nutrition or inadequate education. This key feature is an important component of UHC; indeed, an objective of UHC should be to ensure that health systems develop in a way that is not harmful to other social sectors.

However, one area of confusion in the inter-linkages between financial risk protection and coverage with needed health services under UHC is whether non-use of health services because of financial barriers to access is adequately captured in current measures. This argument is described in detail in Box 1.

**Box 1. Financial Barriers to Access**

UHC requires coverage with financial risk protection and coverage with needed health services side by side. OOP payments contribute to low service coverage rates by deterring people who cannot afford to pay from seeking or continuing care [2],[55],[56]. Financial barriers are also associated with transport costs and lost income involved in seeking care [32],[57],[58]. However, financial barriers are only one of the causes of low levels of service coverage. More fundamentally, if the services are simply unavailable or of poor quality, for example, because of insufficient health workers, medicine, and equipment, people will not be able to obtain the health services they need [59],[60].

Recently it has been suggested that indicators of the lack of financial risk protection capture only the impact of OOP payments on people who use health services but not the fact that others are deterred from seeking care at all. The argument is that common measures of financial risk protection need to be extended to incorporate any non-use of services because of the need to pay [61],[62]. But if both components of UHC are measured at the same time, a complete picture of whether people obtain the services they need and the extent of financial risk protection they encounter in doing so is already provided.

Financial barriers to access may be important to try to measure separately if overall service coverage is not being measured. However, in measuring financial protection and service coverage side by side as part of UHC there is no need to incorporate the impact of financial barriers on utilization into any indicator of financial risk protection. In fact, only highlighting financial barriers to access places an undue importance on them as compared to many other important barriers to access such as cultural norms or geographical inaccessibility.

Another area that causes confusion is related to the component of risk or uncertainty in financial risk protection. The concept of financial risk protection arose from economics and insurance theory [16]–[19]. These disciplines place an importance on explicitly understanding the adverse impacts of uncertainty and its economic value. Theoretically, UHC is also concerned with the adverse impact of uncertainty—the risks that services might be unavailable, of poor quality, or unaffordable in the event that they are needed. However, the indicators that are available to measure financial risk protection do not capture the adverse effects of uncertainty adequately; they only capture the economic hardship encountered because of the lack of financial risk protection. Box 2 describes these points in more detail.

**Box 2. Financial Hardship and Financial Risk Protection**

Financial risk protection in health implicitly involves protection against the financial uncertainty associated with the need to use health services and pay for them. Uncertainty is something that reduces people's peace of mind and wellbeing in itself, and can cause people to change their behaviour, usually in an adverse way. For example, uncertainty about whether necessary health care will be affordable to a family may force them to save large amounts of money that they would have otherwise invested in improving their housing conditions. There is a degree of individual heterogeneity in responding to uncertainty, but by in large, people prefer less rather than more [63].

As discussed earlier, one of the components of UHC is to ensure that no one faces the tough decision of choosing between health care and other necessities. But UHC also goes a step further—it recognizes the “insurance value” of health services being available, of good quality, and affordable in the event that someone needs to use them. People will then have peace of mind that they will be able to access necessary health services when needed without financial hardship. In other words, the uncertainty associated with illness is reduced.

To date, however, it has not been possible to develop generally acceptable ways of measuring the intrinsic value of the reduced uncertainty linked to forms of financial risk protection (or to the knowledge that health services are available and of good quality). The available measures show the effect of the lack of financial risk protection on households. It's also worth keeping in mind that the very core of UHC recognizes that use of needed health services results in better health. Thus there is no need for any additional financial inter-linkage to show that the use of health services contributes to well-being.

Overall, there is a strong synergy between the concepts of coverage with financial risk protection and coverage with needed good quality health services. Indeed, the concepts underlying financial risk protection increase the validity of UHC as a unified health systems goal. The next section examines the specific ways in which financial risk protection is typically measured.

## Common Indicators of Financial Risk Protection

Over the last 15 years, four indicators of financial risk protection have assumed prominence [20],[21]. These indicators are associated with two concepts of financial hardship due to OOP payments, or the absence of financial risk protection: catastrophic health expenditure and impoverishment. We first describe these two concepts along with commonly associated indicators and their relative advantages and disadvantages.

### Catastrophic Expenditures due to Out-of-Pocket Health Payments

Catastrophic health expenditure is the point at which a household's OOP payments are so high relative to its available resources that the household is required to forego the consumption of other necessary goods and services [22]. While the concept is clear, its application has varied in terms of how a household's available resources are calculated and how much of these resources have to be spent on health to cause a catastrophic event. In terms of available resources, catastrophic health expenditures have been defined as health expenditures exceeding a share of either total expenditure, non-food expenditure, or expenditure net of basic food needs. Similarly the threshold at which health payments become catastrophic has ranged from 10% to 40% [22],[23].

Certain considerations are important in choosing the threshold that results in catastrophic expenditure. For example, a relatively high threshold such as 40% increases the likelihood that households incurring discretionary spending on health (e.g., private hospital wards) are not classified as incurring catastrophic expenditures. A high threshold also implies a concern for those who face greater burdens. However, other studies have used a lower threshold (typically applied against total consumption) and some have also assessed the sensitivity of the estimate of financial catastrophe to several different thresholds [24],[25]. Some approaches have even varied the threshold so that it increases as a function of income [26],[27].

Similarly, different choices of the denominator for calculating catastrophic health expenditure, or a household's available resources, are based on different assumptions and priorities. Often a household's non-food expenditure is chosen as the denominator. The idea behind this choice is that a household's food expenditure should not be considered as being part of the resources available to contribute to health. This idea has been taken even further and the standard methodology used by WHO calculates a household's available resources as being expenditure net of basic food spending [7]. Others prefer to use a household's total expenditure as the denominator that is very easy to calculate. However, as expected by economic theory, richer households often tend to spend a higher proportion of their total expenditure on health. As such, the latter measure can be pro-rich, particularly if the threshold for financial catastrophe is set relatively low.

Two indicators measure the concept of catastrophic expenditures (Table 1). The first is the incidence of catastrophic health expenditures, which is a headcount indicator calculated as the proportion of households in a population whose health expenditures exceed this critical point. The second, though less widely used, is the catastrophic overshoot, which captures the extent to which health expenditures exceed the defined threshold [20],[21]. A major advantage of focusing on the concept of catastrophe is that its measurement is across the entire population, that is financial hardship can occur in any population group, including in any income subgroup. Additionally, since the concept is specialized to health and based on a pre-established framework, there is no likelihood of political or societal manipulation of the thresholds or the denominator.

**Table 1:** Key indicators of the concept of catastrophic health expenditure.

Indicator	What It Is Measuring
Incidence of catastrophic health expenditure	Proportion of households in a population who face catastrophic health expenditure
Mean positive catastrophic overshoot	Percentage points by which household spending on health exceeds the threshold for catastrophic health expenditure

### Impoverishment due to Out-of-Pocket Health Payments

The second concept of financial risk protection is the concern that OOP payments can push households below or further below the poverty line. Poverty lines represent a threshold below which even the most basic standard of living is not ensured [28]. OOP payments can be impoverishing in the sense that a household's level of expenditure before making health payments was above the poverty line, but then fell below the poverty line after health expenditures. Importantly, household expenditure in this context includes not only spending in cash, but also spending in kind as well as consumption of self-produced goods, most notably food.

Similar to the way of measuring catastrophic health expenditures, a choice is involved in establishing the poverty line. Absolute poverty lines as well as relative poverty lines exist, but choosing which one to use is largely a value-based decision. An example of an absolute poverty line is the commonly cited dollar a day line, which actually corresponds to the equivalent of US$1.25. This and other absolute poverty lines, which are usually national lines, are calculated on the basis of basic subsistence needs. On the other hand, relative poverty lines are based on the distribution of a specific measure of basic subsistence needs (e.g., basic food expenditure).

The main advantage of an absolute line is that the level of poverty can be easily monitored over time. Its main disadvantage is that it is prone to manipulation by political and societal agents. A relative poverty line, which is determined through actual spending on subsistence needs of different households, does not have the same limitation. Another advantage is that a relative poverty line can account for different patterns in expenditure across countries but it moves in relation to the distribution of poverty in any country [29].

Overall, the choice of poverty line will affect the number of people who are thought to be in poverty; a low poverty line may result in a low rate of impoverishment due to OOP payments. As countries assess their own progress towards UHC, they can use locally defined poverty lines. However, for purposes of international comparisons, it is important to have a common line. One option is to use the US$1.25 or US$2.00 per day per capita (at purchasing power parity) used by the World Bank. Another option is to use a globally defined relative poverty line, such as one based on basic food expenditure as used by the WHO [30],[31].

Two indicators adapted from the general poverty literature to health payments are used to measure this concept (Table 2). The incidence of impoverishment is a headcount measure showing the proportion of households pushed below the poverty line because of OOP payments. A second indicator, again less widely reported, is the increase in depth of poverty, which measures the amount a poor household is pushed further into poverty due to OOP payments.

**Table 2:** Key indicators of the concept of impoverishment due to health spending.

Indicator	What It Is Measuring
Incidence of impoverishment	Proportion of households in a population who fell into poverty due to health spending
Increase in the depth of poverty	Amount by which a household fell further into poverty due to health spending

An important advantage of measuring impoverishment due to OOP payments is that the concept resonates well with policymakers. Indeed, politicians and policymakers from almost all countries in the world are concerned with poverty alleviation. The particular implications of poverty as a multidimensional concept and its linkages with UHC and development goals are explored further in Box 3.

**Box 3. Poverty as a Multidimensional Concept and UHC**

Poverty has long been categorized as a multidimensional construct, rather than just a lack of income or ability to spend [64]–[66]. This definition is different from the mechanical definition of “impoverishment” that has been presented so far in this paper. Under this multi-dimensional thinking, deprivations across different spheres of life, including in education, clean water, and health, can inherently constitute poverty. However, the idea of income (or expenditure) poverty has remained persistent since income poverty is the manifestation of deprivations in goods and services that can be purchased with money. Many social services are at the cross-section of these two ideas: deprivation of social services are inherently causes of non-income poverty and are also manifestations of income poverty since social services can be purchased like any other goods and services.

Income poverty is the concept that has been discussed outside of this box as “impoverishment” in this paper. Indeed, the poverty lines discussed earlier represent how much money is needed so that all purchasable essential needs of an individual are met. “Impoverishment due to OOP payments,” thus occurs when one social service (health) is purchased at the expense of other equally important social services or products.

But there is a very attractive opportunity to construct a measure of “impoverishment” (i.e., income poverty) because of OOP payments that is further aligned with thinking on multi-dimensional poverty. This opportunity is through constructing a poverty line that was based on spending on all essential services and goods except for health. This slight variation in the construction of the poverty line can help further align the concept of financial risk protection and UHC with multi-dimensional poverty. By doing this, manifestations of income-poverty because of OOP payments (i.e., the concept described as “impoverishment” elsewhere in the paper) with the modified poverty line represent the direct adverse impact of health service use on another essential services and products, modulated through income. On the other hand, non-use of health services (the other side of UHC) is a manifestation of non-income poverty either directly or because of lower health status.

In the discussion on the post-2015 development goals, this particular interface of financial risk protection and multi-dimensional poverty merits further consideration. Additionally, it is also possible for other social sectors to take a similar approach. For example, “impoverishment” because of spending on primary education could be calculated on the basis of a poverty line that is net of spending on primary education.

A limitation of the headcount measure of impoverishment is that households that are already below the poverty line will not be accounted for automatically if they are made poorer because of OOP payments. To capture the burden on these households, a measure of the depth of poverty is needed.

### Some Empirical Results

**Figure 1:**
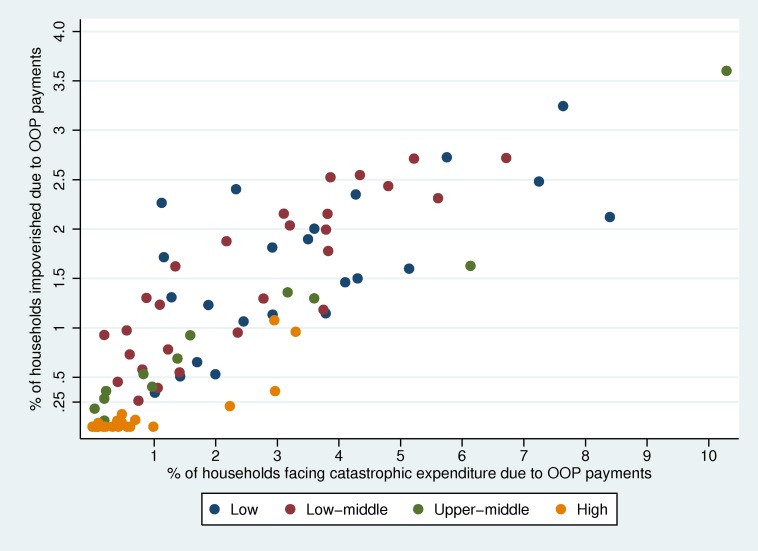
Impoverishment and catastrophic health expenditure headcount by country income.

As described in the previous section, the two concepts of financial hardship (catastrophic health expenditure and impoverishment) measure different aspects of the lack of financial risk protection in health. Figure 1 shows the headcount indicators for the two concepts from 96 household expenditure surveys available to us: although they appear to be correlated across countries, there is some variation. In some countries the incidence of catastrophic health expenditure is much higher than impoverishment, while in others, most commonly in countries where a high proportion of the population lives in near-poverty, the opposite is true. This finding suggests that the two indicators provide different information about the level of financial risk protection. Also, it is worth noting that financial risk protection seems to be better in high-income countries as opposed to low- and middle-income countries, which is related to the development of financial risk protection systems through prepayment and pooling of resources.

**Figure 2:**
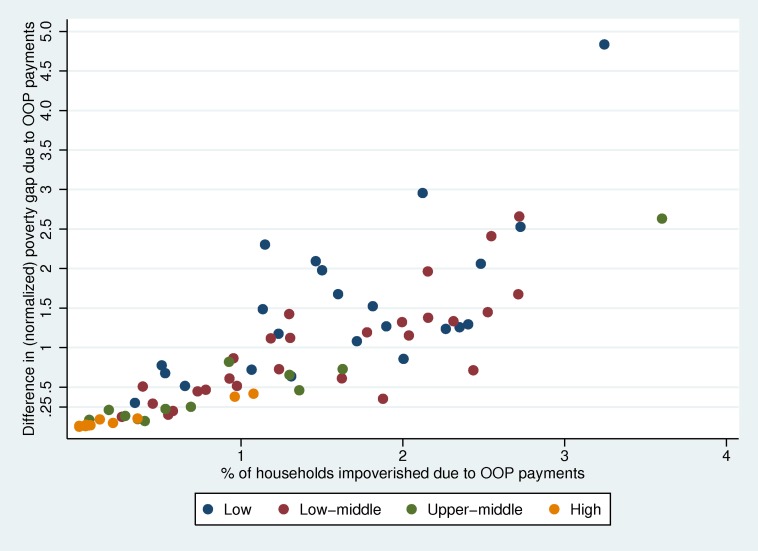
Impoverishment headcount and difference in poverty gap by country income .

Figure 2 plots the impoverishment headcount against the difference in normalized poverty gap for the same datasets. As can be seen, there is a correlation between the poverty headcount and difference in poverty gap due to OOP payments. However, in some countries the increase in depth of poverty due to OOP payments is higher than the average relationship. The opposite is true in some cases. These types of effects are likely to be related to the nature of OOP payments in different settings. But overall, greater information is obtained about the absence of financial risk protection if these two indicators are measured side by side.

**Figure 3:**
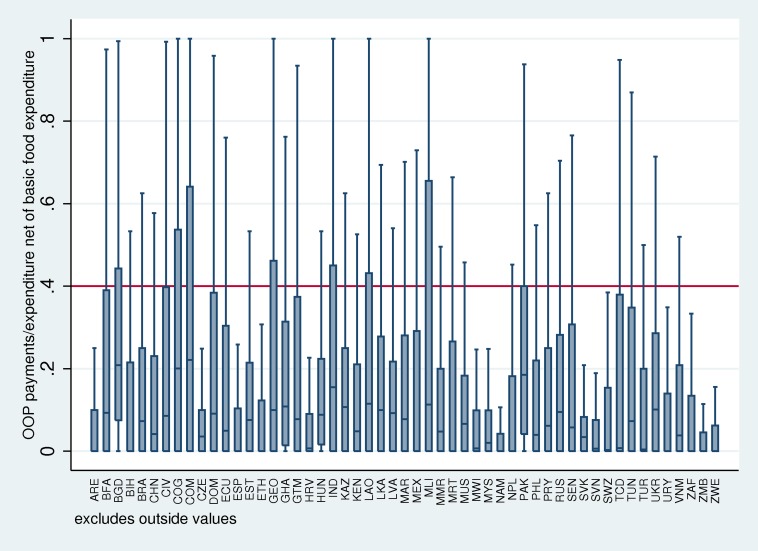
Box plot of OOP payments/expenditure net of basic food expenditure for 53 countries. ARE, United Arab Emirates; BFA, Burkina Faso; BGD, Bangladesh; BIH, Bosnia and Herzegovina; BRA, Brazil; CHN, China; CIV, Côte d'Ivoire; COG, Congo; COM, Comoros; CZE, Czech Republic; DOM, Dominican Republic; ECU, Ecuador; ESP, Spain; EST, Estonia; ETH, Ethiopia; GEO, Georgia; GHA, Ghana; GTM, Guatemala; HRV, Croatia; HUN, Hungary; IND, India; KAZ, Kazakhstan; KEN, Kenya; LAO, Lao People's Democratic Republic; LKA, Sri Lanka; LVA, Latvia; MAR, Morocco; MEX, Mexico; MLI, Mali; MMR, Myanmar; MRT, Mauritania; MUS, Mauritius; MWI, Malawi; MYS, Malaysia; NAM, Namibia; NPL, Nepal; PAK, Pakistan; PHL, Philippines; PRY, Paraguay; RUS, Russian Federation; SEN, Senegal; SVK, Slovakia; SVN, Slovenia; SWZ, Swaziland; TCD, Chad; TUN, Tunisia; TUR, Turkey; UKR, Ukraine; URY, Uruguay; VNM, Viet Nam; ZAF, South Africa; ZMB, Zambia; ZWE, Zimbabwe.

Finally, Figure 3 shows the box plot of OOP payments over household expenditure net of basic food expenditure for 52 countries with the World Health Survey 2003. This methodology is used by WHO for calculating catastrophic health expenditure, where the threshold for catastrophic expenditure is 40%, which is represented by the red line [7]. In the box plot, which contains no outliers, the box represents the inter-quartile range (i.e., 25th–75th percentile of observations). The whiskers on either end of the boxes represent observations falling between 1.5 times the inter-quartile range. This figure demonstrates the idea of the catastrophic overshoot; some households' OOP spending far exceeds the 40% threshold of catastrophic health expenditure. In some countries like Bangladesh, the 75th percentile of the distribution of OOP payments over expenditure net of basic food is above the 40% threshold. In countries like this, the headcount measurement alone will not paint a full picture of the problem of catastrophic health expenditure; an overshoot measurement provides additional information about the severity of the catastrophic spending.

An important critique of these types of indicators is that reliance on OOP payments made at a point in time does not capture other costs such as those related to transportation to health facilities, indirect longer term costs to cope with costs of care, or other health payments by households. While some authors have tried to include these types of payments in their analyses of financial hardship, the methodologies they use are not standard [32],[33]. Expenditure on prepayment for health is also interesting to consider as part of financial risk protection and Box 4 further examines the distinction between OOP payment and pre-payment in more detail.

**Box 4. Prepayment for Health and its Relationship with Financial Risk Protection**

Levels of financial risk protection are clearly related to the way a health system is financed. The more countries rely on prepayment rather than OOP payments, the higher the financial risk protection. There have, however, been some suggestions that household contributions to health through forms of prepayment (largely insurance and taxes) can also cause financial hardship and they should be included in the measurement of financial risk protection [67]. While that is true, we argue that the financial hardship caused by contributions to the health system, or any other system, that are predictable are different to the unpredictable consequences of OOP health payments. Tax rates and compulsory insurance premiums are well established and predictable. Whether they are affordable and fair—that is, whether the poor pay the same amount or proportion of their income as the rich—is important to consider, but is a separate question from protecting people from the unpredictability of payments for health services at the time they get ill.

We recommend that the above indicators be regularly measured if possible, recognizing that the literature has to date largely focused on the two measurements of incidence—of financial catastrophe and of impoverishment. The indicators are relatively straightforward to calculate, easy to understand, and allow for comparative analysis across countries and over time. Previous studies have published the incidence of financial catastrophe and impoverishment because of OOP payments for 89 countries [6],[34]. Their related overshoot and gap indicators are also increasingly being measured and recently developed software allows all four indicators described here to be calculated in a relatively straightforward fashion for any household expenditure survey [35].

## Inequalities in Financial Risk Protection

A central concern of UHC is equity, and thus it is also important to consider who is and who is not protected against the financial hardship imposed by OOP payments. The indicators of financial risk protection are all derived from a household's expenditure, which reflects existing inequalities in income and wealth. Applied studies are also increasingly disaggregating these indicators to examine the hardship imposed on different sub-population groups on the basis of income, wealth, or other socioeconomic characteristics.

Measurement of inequalities in financial risk protection is not always clear-cut. In terms of income, there is evidence of a negative correlation between financial hardship and income [36],[37]. Other studies have found the incidence of financial hardship among people in the poorest quintile is lower than in the rest of the population, often suggesting that this finding is because they either are unable to use health services or because they are already living in poverty [38]. Indeed, the incidence of financial catastrophe can sometimes be higher in higher income quintiles because these people choose to spend more on particular types of health services [39].

Overall, measuring the variation in financial risk protection due to OOP payments across population groups is as important as monitoring the average situation in the population. The relevance of socioeconomic stratifiers used to assess these inequalities may differ across countries according to the main causes of inequality. But some variations, such as those based on income or wealth, place of residence, and sex of household head, are likely to be consistently important across different countries. Other key stratifiers that countries should consider examining may include demographic characteristics of the household, education of the household, and religion and ethnicity of the household. Use of these types of stratifiers is supported by multivariate analyses that show that place of residence (rural/urban or parts of a country), household size or composition (e.g., headed by women, proportion of children or elderly people), and the presence of chronic illnesses, for example, have been associated with increased incidence or severity of financial hardship in different settings [24],[36],[40]–[42]. Understanding the distribution of the burden across sub-population groups will be particularly important as countries implement changes to their health financing policies. However, particular issues related to the monitoring of inequalities in financial risk protection merit some more attention. These are discussed in Box 5.

**Box 5. Further Implications for Measuring Inequalities in the Distribution of Financial Risk Protection**

The headcount measures of impoverishment and financial catastrophe indicate nothing about the severity of the financial hardship. In the former case, the measure also ignores the impact of health payments on households who are already below the poverty line. The difference in normalized poverty gap is often used to capture this as presented in Figure 2. But it has shortcomings from an equity perspective since it gives more weight to the increases in depth of poverty for people just below the poverty line as compared to the poorest people (since OOP payments can never exceed overall consumption). The normalized squared poverty gap, commonly called the squared poverty gap, considers the severity of poverty by giving more weight to the poverty gap of the poorest compared to households just below the poverty line [68]. The difference in squared poverty gap due to OOP payments will also have this property.

A number of composite indicators used in the general poverty literature bring together measures of headcounts and poverty gaps [69]–[72]. Of particular interest could be the Watts index because of its pro-poor properties [73]. The application of these indexes for OOP health payments could be explored although it would be important to find a way of making them easily understood by policymakers.

The alternative to developing composite indicators is a dashboard approach, which presents the four common indicators of the lack of financial risk protection (i.e., incidence of catastrophic health expenditure, catastrophic overshoot, incidence of impoverishment, and difference in poverty gap) and inequalities in them side by side. A challenge for policymakers, as with all sets of indicators, is how to judge if progress is made if the level or equality in one dimension improves, but drops in another. Because of the scarcity of resources for health, this may well happen on the path to UHC.

A way around this might be to add an additional target—that financial risk protection should not decrease for any population group on the path to universal coverage. For this, the Gini coefficient of OOP health payments as a share of a household's non-basic food expenditure could be calculated and compared with the Gini coefficient for the difference in poverty gap due to OOP health payments. Over time changes in these Gini coefficients would show if inequalities in the burden of health payments (rather than only financial catastrophe or impoverishment) had increased.

## Data Requirements for Monitoring Financial Risk Protection

Robust monitoring of financial risk protection requires reliable and periodic household surveys that contain information on health-specific and other expenditures. A recent effort only identified 112 countries that have at least two such surveys at different points in time that would allow the four indicators of financial hardship discussed here to be calculated [43]. But it is important to regularly collect and analyse this information to allow for patterns over time to be assessed; if possible, surveys should be conducted every 2 to 5 years in all countries. Another problem is that general household expenditure surveys are conducted in several countries but are not always specifically analysed for health expenditures [44].

The survey instruments most commonly used to collect health expenditure data differ in aspects such as the recall period, the number of expenditure items covered, and the overall focus of the survey, factors that have shown to influence people's responses. For example, health expenditures reported in surveys (or parts of surveys) focusing on health tend to be higher than those reported in surveys (or sections) where health is only one item under consideration [45]–[47]. Recommendations have been made for greater consistency or more standardised survey instruments to be better able to generate reliable and valid information on financial risk protection as it has not been possible to develop algorithms that adjust health expenditures to account for differences in the survey instrument [46].

Consequently, it would be useful for the organizations that routinely undertake household expenditure surveys to agree on a standard instrument. However, while everyone agrees that standardization is desirable, it is more difficult to convince people to use someone else's “standard instrument” rather than their own. Additionally, with an increasing focus on UHC, the same surveys should also cover utilisation of health services where possible. Lastly, the question exists whether there should be a direct linkage of health services and financial risk protection for these services using survey data. We do not recommend this option, but discuss the idea further in Box 6.

**Box 6. Financial Risk Protection and the Benefit Package**

Improving financial risk protection is not just influenced by health systems financing arrangements, but also by choices about a benefit package where one is specified. For example, if countries finance a benefits package that focuses on high cost items, or items that may be used frequently even if they are relatively low cost, they might provide greater financial risk protection than other types of benefits packages. This is one reason why Verguet and colleagues have proposed to develop a form of “extended cost-effectiveness analysis” that takes into account the impact of a proposed intervention not just on health but also on financial risk protection [74]. This form of analysis, however, is very much in its infancy.

The other side of the coin is that people might incur OOP payments on items that are not in the benefits package. This is inevitable where benefits packages are shallow, as in most lower-income countries. In higher-income countries with extensive packages, however, people might choose to spend on items outside the package, possibly on items that are not strictly necessary to promote or maintain their health. The question then arises of whether this type of expense should be excluded from the estimates of financial catastrophe and impoverishment. This raises some ethical questions; for example, should we consider need as perceived by an individual or by the medical profession? In principle, we should probably exclude expenditure on non-essential services if they could be identified, but this poses a significant measurement challenge. The household surveys used to collect information on OOP payments do not provide enough detail to identify the types of services on which people spend. The few researchers who looked at this question have found in some countries richer households suffer more financial catastrophe than others but they spend more on hospitalization and dental care, while poorer households who face financial hardship spend more on medicines and outpatient expenditure, and there is a suspicion that some of the expenditure of richer households is not strictly necessary, but it is difficult to prove [38],[75].

## Discussion

Measuring coverage of needed health services side by side with the extent of financial risk protection in health and inequalities in both provides a complete picture of who can use the health services they need and the financial consequences of this use. These are the critical components of UHC. This paper outlines the four indicators of the lack of financial risk protection: two are now widely used and two others are increasingly being used to show average levels and inequalities on the path to UHC.

In interpreting the information provided by this type of analysis, however, a number of qualifications need to be highlighted. Firstly, the common measures of financial hardship are not well-suited to understand the long term implications on household economic well-being [48],[49]. We can, for example, identify the number of households pushed into poverty at various points in time, but it is not possible to tell what happens to them subsequently. If they manage to rebound shortly afterwards then perhaps there would be less concern than if they are trapped in poverty for long periods of time. Exploring these issues requires frequent panel data and the ability to track individual households over time, which is expensive and administratively complex.

Related to this complexity is the fact that most household expenditure surveys reveal little about how households cope with health shocks and the resulting financial consequences [50],[51]. For example, some surveys contain questions about whether households financed their health expenditures through savings, selling assets, or borrowing. While the responses can provide some insights, they are often difficult to interpret because of the different ways savings are used in different countries [52]. More detailed research comparing the incomes, consumption, savings, investments, and wealth of people with and without health shocks, which can adjust for confounders, is required to understand fully how households cope.

There are also linkages between social protection and financial risk protection. In addition to immediate financial consequences, households encounter problems such as loss of employment or wages because of taking time off work [33]. Financial risk protection is thus just a component of even broader social protection that is needed to ensure that there are no adverse consequences associated with using needed health services. However, these broader research and policy questions lie largely outside the health sector and the boundaries of UHC.

Overall, the number of studies focusing on financial risk protection in health has increased substantially over the last decade, and some studies have already been instrumental in stimulating policy changes in countries such as Mexico and Thailand [53],[54]. Accordingly, it will be important not only to continue developing new methodologies, but also to find ways to make the results intuitively understandable to decision makers.

## Conclusions and Recommendations

With an increasing focus on UHC, there is a clear need to better understand its underlying concepts and practical methods of assessing their progress. Existing ways to measure financial risk protection provide useful insights into the financial hardship caused by accessing needed health services. For countries to benefit from sound policy making, regular monitoring of both the levels of and inequalities in key indicators of financial risk protection are needed. (Box: recommendations).

### Recommendations

- At the country level, routinely measure the incidence of financial catastrophe and impoverishment and associated inequalities to understand if the situation is improving. Where possible, also measure the catastrophic overshoot and the difference in the poverty gap for further insights.
- Where possible, standardise survey instruments and data on the use of health services.

## Abbreviations

OOP: out-of-pocket
UHC: universal health coverage
